# A statistical method for adjusting covariates in linkage analysis with sib pairs

**DOI:** 10.1186/1471-2156-4-S1-S51

**Published:** 2003-12-31

**Authors:** Colin O Wu, Gang Zheng, Eric Leifer, Dean Follmann, Jing-Ping Lin

**Affiliations:** 1 Office of Biostatistics, DECA, National Heart, Lung, and Blood Institute, 2 Rockledge Center, Bethesda, Maryland, USA

## Abstract

**Background:**

We propose a statistical method that includes the use of longitudinal regression models and estimation procedures for adjusting for covariate effects in applying the Haseman-Elston (HE) method for linkage analysis. Our methodology, which uses the covariate adjusted trait, contains three steps: a) modelling the covariate-adjusted population means of quantitative traits through regression; b) estimating the value of covariate-adjusted quantitative traits; and c) evaluating the linkage between the adjusted trait values and the markers based on alleles shared identically by descent.

**Results:**

We applied our adjusted HE method and the standard HE method in S.A.G.E. to the sib-pair subset of the Framingham Heart Study distributed by Genetic Analysis Workshop 13 with systolic blood pressure as the quantitative trait. Both methods gave similar patterns for the LOD scores, and exhibited highest multipoint LOD scores near location 70 cM of chromosome 12.

**Conclusion:**

The adjusted HE method has two major advantages over the standard HE method used in S.A.G.E.: a) it has the capability to handle longitudinal data; b) it provides a more natural approach for adjusting the repeatedly measured covariates from each subject.

## Background

Let *X*_1*j *_and *X*_2*j *_be the observed trait values for the first and second sibs in a cross-sectional study, and let *Y*_*j *_= (*X*_1*j *_- *X*_2*j*_)^2 ^be the squared trait difference in the *j*^th ^sib pair. The Haseman-Elston (HE) method [1] assumes that

*X*_*ij *_= μ + *g*_*ij *_+ *e*_*ij*_,     (1)

where *i *= 1,2, μ is the overall mean trait value, and *g*_*ij *_and *e*_*ij*_, which are independent and have mean zero, represent the genetic and environmental effects, respectively, on the *i*^th ^sib of the *j*^th ^sib pair [denoted by the (*i, j*)^th ^sib hereafter].

Suppose that, in addition to the genetic and environmental effects, the phenotype *X*_*ij *_is also affected by a set of *p *covariates. Let  be the value observed for the *l*^th ^covariate in the (*i, j*)^th ^sib (*j *= 1,2; *i *= 1,...,*n*; *l *= 1,...,*p*). Given the proportion of genes identical by decent (IBD), π_*j *_for the *j*^th ^sib pair and covariates  for the (*i, j*)^th ^sib, Elston et al. [2] described the genetic and covariate effects on the phenotype through the linear model



where  is a transformed covariate determined by  and , β_0 _describes the linkage between the phenotype and the marker alleles, and β_1_,...,β_*p *_represents the covariate effects. Estimation and inference procedures based on equation (2) have been incorporated into the software package SIBPAL in S.A.G.E. [3].

In practice, however, there are two potential limitations for the method based on equation (2). First, since the model and its estimation procedures currently used in S.A.G.E. [3] are designed only for cross-sectional studies, they are generally not suitable for longitudinal studies where the data are repeatedly obtained over time. Second, in some situations it may not be appropriate to select  as the original covariates observed in the data set, so that an adequate implementation of equation (2) depends on choosing a sensible transformation applied to the original covariates. To overcome these shortcomings, we propose in this paper an alternative sib-pair approach that can be applied to longitudinal studies and that adjusts the covariates prior to linkage analysis. The approach of adjusting covariate prior to linkage analysis has been previously considered in the literature under some different contexts (e.g., Amos [4] and Suh et al. [5]). Our method is focused on combining the HE method with statistical procedures for covariate adjustment using the generalized estimating equations (GEE) and within-cluster resampling [6-8] and contains three main steps: a) modelling the covariate-adjusted population means of quantitative traits through regression; b) estimating the covariate-adjusted quantitative traits; and c) evaluating the linkage between the adjusted trait values and the marker alleles shared IBD. The objective is to link phenotype with the proportion of genes shared IBD using only the trait values after removing the influences of the covariates that are unrelated to the genes. Numerical computations of our method can be easily implemented using the existing statistical and genetic software packages, such as SAS (SAS/STAT Software [9]) and SIBPAL (S.A.G.E. [3]).

Applying our method and the standard HE method (equation (2)) in S.A.G.E. to the Genetic Analysis Workshop 13 Framingham Heart Study data, we found that both methods gave similar results for cross-sectional analyses using only the data from one visit, and exhibited highest multipoint LOD scores near location 70 cM of chromosome 12 for the longitudinal analyses. Our method is more natural at handling the longitudinal data and has generally higher peak multipoint LOD scores than the standard HE method.

## Methods

### Modelling the covariates

For cross-sectional studies, we assume that the covariates are not affected by the genes and generalize equation (1) to



where  is the expectation of *X*_*ij *_given the covariates  and the unknown (*p *+ 1) dimensional parameter ψ, and  is the covariate adjusted trait for the (*i, j*)^th ^sib. When μ(·;·) is a simple linear link function, the value of the covariate adjusted phenotype is



where ψ = (ψ_0_,...,ψ_*p*_) are the linear coefficients.

For longitudinal studies, let *n*_*ij *_be the number of repeated measurements and *T*_*ijk*_, *k *= 1,...,*n*_*ij*_, be the time of the *k*^th ^measurement for the (*i, j*)^th ^sib. Assume that the conditional expectation of *X*_*ijk *_given the covariate vector  is , which is determined by the (*p *+ 2) dimensional parameter ψ and holds for all the measurement points. Assume further that the gene effects on the quantitative trait values are the same for all the measurement points. By adjusting the time-dependent covariates , we have the longitudinal model



Then the linear model with coefficients ψ_0_,...,ψ_*p*+1 _is



### Methods for cross-sectional studies

Using the covariate adjusted squared trait difference , the same derivation as in Table 1 of HE [1] shows that



and the problem of linkage detection reduces to testing the statistical hypothesis of β = 0 (no linkage) versus the one-sided alternative β < 0 (linkage). If there were a consistent estimate  for ψ, then  and  can be estimated by  and , respectively. The hypotheses β = 0 and β < 0 can be tested using the standard HE procedure with  as the observed squared trait difference.

Since the sibs in the same family are correlated, there are two approaches that can be used to estimate ψ. The first is to treat the families as independent subjects and apply the existing longitudinal methods, such as the generalized estimating equations (GEE, e.g., [6,10]) to the observed traits. The second approach is within-cluster resampling [7,8], which first generates multiple independent resampled data sets by randomly drawing one sib from each family, estimates ψ from each resampled data set using the existing estimation methods for independent cross-sectional data, and finally computes  by averaging the estimates computed from all the resampled data. When the number of families is large, both approaches are expected to lead to consistent estimates.

### Methods for longitudinal studies

We assume here that both sibs in a pair have the same number of repeated measurements, i.e., *n*_1*j *_= *n*_2*j *_= *n*_*j *_for all *j*. The adjusted squared trait difference at the *k*^th ^measurement for the *j*^th ^pair is , and averaging over all the measurements, the average adjusted squared trait difference for the *j*^th ^pair is . Then equation (7) continues to hold if  were replaced by , and, consequently, the linkage between the phenotype and the marker loci can be detected by testing β = 0 against β < 0. If there were a consistent estimate , we could estimate  by , where



and the standard HE procedure would be implemented using  as the observed squared trait difference for the *j*^th ^pair.

The estimation of ψ is now affected by two sources of correlations: the correlation between the repeated measurements within a sib and the correlations between sibs within the same family. Ideally, it is possible to model these correlation structures and incorporate these correlation models into an established longitudinal estimation procedure, such as GEEs. But the potential bias and variability that may be associated with the possible model misspecifications of this approach have not been well studied. As a practical alternative, we suggest the following three-step within-cluster resampling procedure: a) randomly sample one sib from each family; b) estimate ψ from the resampled data using GEE; and c) repeat the above steps multiple times and calculate  by averaging the estimates from all the resampled data sets. When the number of families is large,  is expected to be a consistent estimate of ψ [7,8].

### Choosing correlation structures in GEE

An important issue in obtaining an appropriate value for a covariate-adjusted phenotype is to select a suitable covariate structure for implementing the GEE procedure in SAS or other statistical programs. For cross-sectional studies, equation (3) is equivalent to



where  is the marginal mean of *X*_*ij *_given  and ψ, and ε_*ij *_is the error term determined by the gene and the environment. This model falls into the framework of marginal models of Diggle et al. [[6], Ch. 7]. When GEE is used for estimating the unknown parameter ψ of the marginal component, a suitable covariance structure that can be generally applied with the PROC MIXED procedure in SAS [9] is the "compound symmetry" model. Explicit mathematical expression of the compound symmetric covariance structure is given by Verbeke and Molenberghs [[10], page 117]. Other covariance structures such as the ones described in Diggle et al. [[6], Ch. 5] may also be considered when additional details about the error term ε_*ij *_are available.

For longitudinal studies, we can apply the GEE procedure with the same compound symmetric covariance structure as in Verbeke and Molenberghs [[10], page 117] to the within-cluster resampled data with the marginal model



so that consistent estimate  for ψ in the marginal component can be obtained. Since only one sib in a family is randomly chosen in the resampled data, compound symmetry is an adequate assumption for the correlation structure here.

### Comparison with the standard HE method

Theoretically, our covariate-adjusted linear model (equation (7)) is equivalent to the standard HE model (equation (2)) if proper transformations for the original covariates are used. To see this, we compare these models for the cross-sectional data, as similar arguments can be made for the case with longitudinal data. Let μ_*ij *_= . Assume, for simplicity, that the covariates  are non-random. (The same conclusion for the random covariate case can be similarly derived by taking conditional expectations given these covariates, assuming that  are independent of π_*j*_.) Direct calculation using the definitions of *Y*_*j *_and  then shows that

*E*( | π_*j*_) = *E*(*Y*_*j *_| π_*j*_) - (μ_1*j *_- μ_2*j*_)^2 ^= α + βπ_*j*_.     (8)

When μ_*ij *_is a simple linear link function as given in the second term at the right side of the equation (4), equation (8) is clearly a special form of equation (2) with the covariates at the right side of equation (2) taken to be quadratic and cross-product transformations of the original covariates. When μ_*ij *_is a nonlinear function of the original covariates, suitable covariate transformations have to be used to make equation (2) equivalent to equation (8). In practice, however, it is often difficult to determine what meaningful covariate transformation should be used in equation (2). In our view, the appeal of equation (7) or its equivalent, equation (8), compared to the standard HE method is that the variations on phenotype contributed by the nuisance factors (covariates unaffected by genes) can be naturally modelled and adjusted before analysing the gene effects.

## Data

The second generation data from the Framingham Heart Study distributed by the Genetic Analysis Workshop 13 contain 482 multi-sib families from a total of 576 nuclear families from 330 pedigrees. We used all the possible sib pairs from these families and the repeated measurements from all their visits. For the purpose of illustration, we specified systolic blood pressure (SBP) as the quantitative trait, and the subject's age (in years), gender (0 for female, and 1 for male), and average daily alcohol consumption (in milliliters) as the covariates of interest. The subjects had up to five visits during the study, but not all the subjects completed all five visits. Among a total of 1672 subjects that were included in the data set, the numbers of subject who were measured at visits 1 through 5 were 1649, 1393, 1402, 1439, and 1377, respectively.

## Implementation

### Adjusted HE method

We assumed that the mean SBP conditioning on the subject's gender, and age and drinking level (ml/day) at the visit was determined by

μ (gender, age, drink; ψ) = ψ_0 _+ ψ_1 _× gender + ψ_2 _× age + ψ_3 _× drink.

Using the three-step resampling procedure, we generated 1000 independent resampled data sets from the entire sib-pair data that included all the observed visits, and computed  by averaging the GEE estimates obtained from the resampled data sets using the compound symmetric covariance structure. For each sib pair, we used the visits in which the measurements were available for both sibs, estimated the covariate adjusted SBP at the *k*^th ^visit by



and computed the adjusted squared SBP difference by



where *n*_*j *_is the number of common visits for both sibs of the *j*^th ^sib pair. We then performed the genome scan using the adjusted squared SBP difference and the existing HE procedure in S.A.G.E. [3].

For comparison, we performed the cross-sectional analysis using the adjusted HE method as above on data from visit 1. This visit was used because it had the measurements for most of the participating subjects. Since only one visit was used, the phenotype for each sib pair was simply the squared difference of the sibs' covariate adjusted SBP.

### Standard HE method

The current HE procedure in S.A.G.E. is not capable of handling the longitudinal data from multiple visits. In order to transform the measurements from multiple visits into the data structure that can be taken by S.A.G.E., we used for each subject the average values of his/her SBP, age, and drinking level over his/her available visits, and fitted the model (2) using the phenotype

*Y_j _*= [(average SBP)_1*j *_- (average SBP)_2*j*_]^2^

and the covariates



and  where (*average A*)_*ij *_denotes the averaged value of *A *for the (*i, j*)^th ^sib over his/her repeated measurements.

Although the above approach is ad hoc, it is a practical way for transforming repeated measurements to the data framework currently acceptable by S.A.G.E., and should be generally acceptable if the data are balanced in the sense that there are very few missing values in the data set. However, when the data are unbalanced, the approach of averaging out observed measurements over different time points may lead to potential bias for the analysis. One approach for handling the imbalance caused by missing data is to combine the above approach with multiple imputation. But its adequacy under the current setting deserves further investigation.

To compare the standard HE method with our adjusted HE method in cross-sectional data, we fitted the standard HE model using only the data from visit 1 with the squared trait difference *Y*_*j *_= (*SBP*_1*j *_-*SBP*_2*j*_)^2^, and covariates



The same procedure can be repeated for cross-sectional data from other visits. But, since the results from the cross-sectional analyses do not dramatically differ from one another, we only included the results for visit 1 in the presentation.

## Results

### Longitudinal analyses

**Figure 1:**
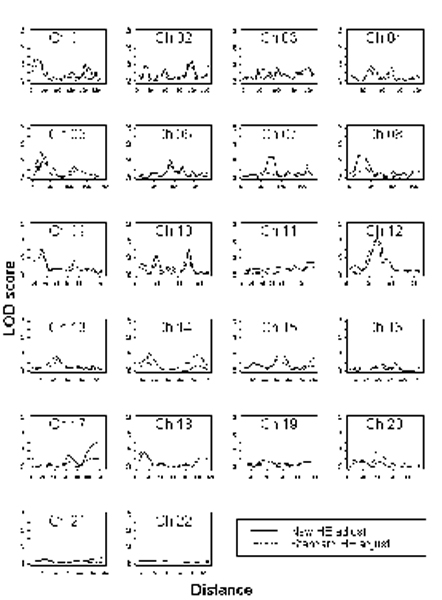
**LOD scores based on longitudinal analyses **The solid curves represent the multipoint LOD scores obtained after adjusting for gender, age, and drinking level using the adjusted HE method. The dashed curves represent the multipoint LOD scores obtained after adjusting for gender, age, and drinking level using the standard HE method in S.A.G.E.

Figure 1 shows the multipoint LOD scores based on the adjusted HE method and the standard HE method for all 22 chromosomes. Both methods exhibit similar general patterns for the LOD scores. The largest LOD scores are 2.3 to 2.6 for the adjusted HE method and the standard HE method, respectively, both appear near position 70 cM of chromosome 12. The adjusted HE method also shows a few higher LOD score peaks than the standard HE method at other locations. But all these LOD scores are smaller than 1.6.

### Cross-sectional analyses

**Figure 2:**
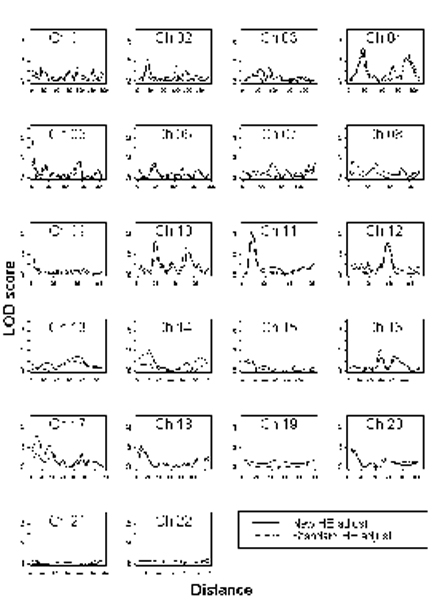
**LOD scores based on data from visit 1 **The solid curves represent the multipoint LOD scores obtained after adjusting for gender, age, and drinking level using the adjusted HE method. The dashed curves represent the multipoint LOD scores obtained after adjusting for gender, age, and drinking level using the standard HE method in S.A.G.E.

Figure 2 shows the multipoint LOD scores for all 22 chromosomes based on the visit 1 data. The LOD scores under both methods have similar patterns. Compared with the longitudinal analysis, the LOD score peaks at chromosome 12 are slightly smaller and their locations are slightly shifted; however, several peaks, such as the ones in chromosomes 4 and 11, do not appear in the longitudinal analyses.

## Discussion

We have proposed a new statistical method for adjusting the effects of covariates for the HE regression when longitudinal data are present. Compared with the linear covariate adjustment in S.A.G.E., our method has two advantages. First, it can handle repeated measurements in a longitudinal study using the existing software such as SAS and S.A.G.E. Second, it adjusts the covariates prior to linkage analysis in a natural way. There are a number of possibilities to generalize our approach. One option is to replace the additive assumptions on the gene and environmental effects in equation (5) by terms that allow for interactions. Other options may aim at modelling and estimating the covariate effects based on more general parametric and nonparametric statistical models. These generalizations require redeveloping the relationship between the trait difference and the proportion of genes IBD.
